# CoVaccine HT™ Adjuvant Potentiates Robust Immune Responses to Recombinant SARS-CoV-2 Spike S1 Immunization

**DOI:** 10.3389/fimmu.2020.599587

**Published:** 2020-10-30

**Authors:** Brien K. Haun, Chih-Yun Lai, Caitlin A. Williams, Teri Ann S. Wong, Michael M. Lieberman, Laurent Pessaint, Hanne Andersen, Axel T. Lehrer

**Affiliations:** ^1^ Department of Tropical Medicine, John A. Burns School of Medicine, University of Hawaii, Honolulu, HI, United States; ^2^ Cell and Molecular Biology Graduate Program, John A. Burns School of Medicine, University of Hawaii, Honolulu, HI, United States; ^3^ BIOQUAL, Inc., Rockville, MD, United States

**Keywords:** adjuvant, recombinant subunit, COVID-19, immunogenicity, rapid response, SARS-CoV-2

## Abstract

The current COVID-19 pandemic has claimed hundreds of thousands of lives and its causative agent, SARS-CoV-2, has infected millions, globally. The highly contagious nature of this respiratory virus has spurred massive global efforts to develop vaccines at record speeds. In addition to enhanced immunogen delivery, adjuvants may greatly impact protective efficacy of a SARS-CoV-2 vaccine. To investigate adjuvant suitability, we formulated protein subunit vaccines consisting of the recombinant S1 domain of SARS-CoV-2 Spike protein alone or in combination with either CoVaccine HT™ or Alhydrogel. CoVaccine HT™ induced high titres of antigen-binding IgG after a single dose, facilitated affinity maturation and class switching to a greater extent than Alhydrogel and elicited potent cell-mediated immunity as well as virus neutralizing antibody titres. Data presented here suggests that adjuvantation with CoVaccine HT™ can rapidly induce a comprehensive and protective immune response to SARS-CoV-2.

## Introduction

The outbreak of 2019-novel coronavirus (SARS-CoV-2), the etiological agent of Coronavirus Disease 2019 (COVID-19), began in Wuhan, China in late 2019 and quickly spread across the globe causing epidemics on every continent except Antarctica in under four months. This virus has caused more than 33 million cases and over 1 million deaths worldwide (as of 9-29-20) (1). SARS-CoV-2 is highly transmissible during both the pre-symptomatic and acute symptomatic phases and the infection fatality rate has been reported as high as 3.4% (2). COVID-19 often develops into severe illness, including pneumonia. Currently, there are no licensed vaccines or effective therapeutic strategies available to treat COVID-19. Evidence indicates that high titres of antibody targeting the Spike protein may neutralize virus, a concept which carries credence for the closely related SARS-CoV (3–5). Vaccine platforms at the forefront of development are mRNA-based, DNA-based, virally vectored (replication competent or incompetent), as well as recombinant protein subunits (6, 7). Many vaccine candidates may require adjuvantation to induce robust immune responses and rapidly induce high antibody titres. However, no consensus is established on an optimal adjuvant that best induces protective immunity to SARS-CoV-2.

In order to investigate which adjuvants induce a strong humoral response, our group has formulated protein subunit vaccine candidates using a recombinant SARS-CoV-2 Spike subdomain 1 (S1) protein, obtained from Sino Biological, Inc., adjuvanted with either CoVaccine HT™ or Alhydrogel. The former is a proprietary adjuvant and the latter is an FDA approved adjuvant used in several FDA licensed vaccines. CoVaccine HT™ is an oil-in-water (O/W) emulsion of hydrophobic, negatively-charged sucrose fatty acid sulphate esters with the addition of squalane (8, 9) whereas Alhydrogel is a colloid of aluminium hydroxide which binds protein to facilitate antigen recognition and thus, improve the immune response (10). The mechanism of action of Alhydrogel remains somewhat elusive, however this adjuvant likely interacts with NOD like receptor protein 3 (NLRP3) but does not interact with TLRs (11). This difference in cellular activation can account for the disparities seen between the use of CoVaccine HT™ and Alhydrogel presented here. The stabilized oil in water emulsion functions by generating a response skewed towards a T-helper type 1 cell (T_h_1) direction which can in turn sustain CD8 T cells capable of mitigating viral infection (12). This adjuvant is also capable of inducing T cell differentiation to T-follicular helper (T_fh_) cells which is evident through immunoglobulin class switching to IgG2a (13). In concert, these cellular responses enhance the humoral response evidenced by the overall higher titres of IgG (13).

CoVaccine HT™ also offers an advantage in comparison to Alhydrogel regarding particle size. Alhydrogel particles typically fall within the range of 1-10 microns (14) whereas CoVaccine HT™ is typically showing droplet sizes around 130 nm (15, 16). Smaller particle sizes offer increased stability and enhanced adjuvanticity and in comparison, particle sizes of other commercial stable O/W emulsion adjuvants (MF59 and AS03) are in the range of 160 nm (17). These O/W emulsion adjuvants utilize squalene, a shark fat derived product (9, 18, 19). The use of squalane in CoVaccine HT™ as a plant-derived product may be advantageous due to availability, reduced regulatory burden, and potentially also ideologically to the population being immunized. In summary, CoVaccine HT™ could provide a distinct advantage over Alhydrogel as the more conventional adjuvant choice.

Here we tested the immunogenicity of SARS-CoV-2 Spike S1 proteins adjuvanted with either CoVaccine HT™, Alhydrogel, or phosphate buffered saline (PBS) in BALB/c mice. We assessed overall antibody titres, immunoglobulin subclass diversity, cell mediated immunity, and in-vitro neutralization of wild-type SARS-CoV-2 virus. We demonstrate that CoVaccine HT™ elicits rapid humoral responses, increased subclass diversity, more interferon gamma (IFN-γ) production, and higher neutralizing antibody titres than the other adjuvants. Collectively, CoVaccine HT™ may be advantageous over other adjuvants for a SARS-CoV-2 vaccine.

## Methods

### Vaccination and Serum Collection

BALB/c mice (7–8 weeks of age, male and female) were immunized twice, three weeks apart, intramuscularly (IM) with 5 µg of SARS-CoV-2 Spike S1 (Sino Biological 40592-V05H) protein with or without adjuvants, or adjuvant alone, using an insulin syringe with a 29-gauge needle. The adjuvants used were CoVaccine HT™ (Protherics Medicines Development Ltd, London, United Kingdom), or 2% Alhydrogel adjuvant (InvivoGen, San Diego, CA). Sera were collected by tail bleeding at 2 weeks post-immunization or cardiac puncture for terminal bleeds. An additional serum sample was collected by cardiac puncture at day 28 along with splenocytes from three animals in the Spike S1 + CoVaccine HT™ (S1+CoVac) and S1 + Alum group, and two animals in the S1+PBS group. Mouse studies were conducted with approval from the Institutional Animal Care and Use Committee at the University of Hawaii. All ethical regulations were complied with regarding animal work.

### Serological Immunoglobulin Assays

Carboxylated, infrared dyed, magnetic microspheres (Mag-Plex™-C) were obtained from Luminex Corporation (Austin, TX, USA) and coupled to each antigen used in the study as previously described (20, 21). For coupling, a two-step carbodiimide process recommended by Luminex was used to link 10 µg of purified proteins to the surface of 1.25×10^6^ microspheres. Microspheres were also coupled with bovine serum albumin as a negative control. SARS-CoV-2, SARS-CoV, and MERS-CoV specific immunoglobulin antibody titres in mouse sera were measured using a microsphere immunoassay as previously described with some minor alterations (13, 22, 23). Briefly, microspheres coupled to his-tagged Spike S1 proteins of SARS-CoV-2, SARS-CoV, or MERS-CoV (Sino Biological 40591-V08H, 40150-V08B1, & 40069-V08H, respectively), and control beads coupled to bovine serum albumin (BSA) were combined and diluted in MIA buffer (PBS-1% BSA-0.02%Tween20) at a dilution of 1/200. Multiplex beads (at 50 μl containing approximately 1,250 beads of each type) were added to each well of black-sided 96-well plates (Greiner). 50 μl of diluted serum were added to the microspheres in duplicate and incubated for 3 h on a plate shaker set at 700 rpm in the dark at 37°C. The plates were then washed twice with 200 μl of MIA buffer using a magnetic plate separator (Millipore Corp., Billerica, MA). 50 μl of red-phycoerythrin (R-PE) conjugated F(ab’)2 fragment goat anti-mouse IgG specific to the Fc fragment (Jackson ImmunoResearch, Inc., West Grove, PA) were added at 1 µg/ml to the wells and incubated for 45 min. Antigen-specific IgG subclass titres were determined using mouse antisera at a 1:1,000 dilution. Detection antibodies were subclass specific goat anti-mouse polyclonal R-PE-conjugated antibodies (Southern Biotech) used at a 1:200 dilution. The plates were washed twice, as described above, and microspheres were then resuspended in 120 µl of drive fluid (MilliporeSigma) and analysed on the MAGPIX Instrument (MilliporeSigma). Data acquisition detecting the median fluorescence intensity (MFI) was set to a minimum of 50 beads per spectral region. Antigen-coupled beads were recognized and quantified based on their spectral signature and signal intensity, respectively. Assay cut-off values were calculated first by taking the mean of technical duplicate values using the average MFI (indicated as a dashed black line) from the adjuvant only control group. Cut-offs were generated by determining the mean MFI values plus three standard deviations as determined by Microsoft Office Excel program. Graphical representation of the data was done using Prism, Graphpad Software (San Diego, CA).

### Plaque Reduction Neutralization Test (PRNT)

A PRNT was performed in a biosafety level 3 facility (at BIOQUAL, Inc.) using 24-well plates. Mouse sera pooled from individual mice within each group, were diluted to 1:10, and a 1:3 serial dilution series was performed 11 times. Diluted samples were then incubated with 30 plaque-forming units of wild-type SARS-CoV-2 (USA-WA1/2020, BEI Resources NR-52281) in an equal volume of culture media (DMEM-10% FBS with gentamicin) for 1 h at 37°C. The serum-virus mixtures were added to a monolayer of confluent Vero E6 cells and incubated for 1 h at 37°C in 5% CO_2_. Each well was then overlaid with 1 ml of culture media containing 0.5% methylcellulose and incubated for 3 days at 37°C in 5% CO_2_. The plates were then fixed with methanol at -20°C for 30 min and stained with 0.2% crystal violet for 30 min at room temperature. Neutralization titres were defined as the highest serum dilution that resulted in 50% (PRNT_50_) and 90% (PRNT_90_) reduction in the number of plaques.

### Preparation of Mouse Splenocytes and FluoroSpot Assay

Mouse spleens were harvested at day 7 after the second dose, minced, passed through a cell strainer, and cryopreserved after lysis of red blood cells. Cellular immune responses were measured by IFN-γ FluoroSpot assay according to the manufacturer’s instructions (Cat. No. FSP-4246-2 Mabtech, Inc., Cincinnati, OH). Briefly, splenocytes were rested at 37°C, in 5% CO_2_ for 3 h after thawing to allow removal of cell debris. A total of 2.5 x 10^5^ cells per well in serum-free CTL-Test™ medium (Cellular Technology Limited, Shaker Heights, OH) were added to a 96 well PVDF membrane plate pre-coated with capture monoclonal antibodies and stimulated for 40 h with peptides, PepTivator^®^ SARS-CoV-2 Prot_S1 peptide pool consisting of 15-mer peptides with 11 amino acids overlapping, covering the N-terminal S1 domain of the Spike protein of SARS-CoV-2 (Miltenyi Biotec, Auburn, CA) at 0.2 μg/ml and 0.5 μg/ml per peptide, or medium alone. Splenocytes at (5 x 10^4^ per well) were incubated with PMA (0.01 μM)/Ionomycin (0.167 μM) cocktail (BioLegend, San Diego, CA) as a positive control. The tests were set up in duplicates, and the costimulatory anti-CD28 antibody (0.1 µg/ml) was added to the cells during the incubation. Plates were developed using specific monoclonal detection antibodies and fluorophore-conjugated secondary reagents. Finally, plates were treated with a Fluorescence enhancer (Mabtech) to optimize detection and then air-dried. The spots were enumerated using the CTL ImmunoSpot^®^ S6 Universal Analyzer (Cellular Technology Limited, CTL, Shaker Heights, OH), and the number of antigen specific cytokine-secreting spot forming cells (SFCs) per million cells for each stimulation condition was calculated by subtracting the number of spots detected in the medium only wells.

### Statistics

All statistical tests were performed using GraphPad Prism software version 8.0 (GraphPad software, San Diego, CA).

## Results

### Murine Immunization With SARS-CoV-2 Spike S1 Proteins

**Figure 1:**
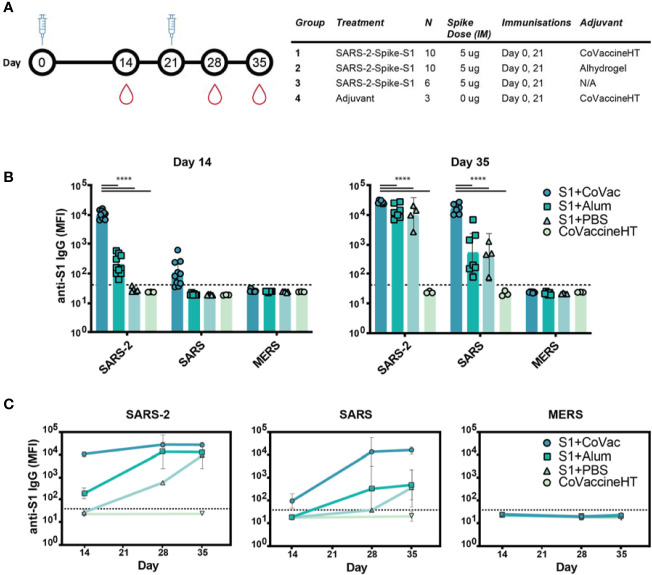
Immunogenicity and specificity to SARS-CoV-2 S1 immunization. **(A)** Timeline schematic of BALB/c immunizations and bleeds with a table detailing the study design. **(B)** Median fluorescence intensity (MFI) of serum antibodies from each group binding to custom magnetic beads coupled with Spike S1 proteins from either SARS-CoV-2 (SARS-2), SARS-CoV (SARS), or MERS-CoV (MERS) on day 14 and 35. **(C)** Antibody reactivity to SARS-2, SARS, and MERS antigens throughout the study. Graphs in **(B, C)** are on a logarithmic scale representing geometric mean MFI responses with 95% confidence interval (CI). The dashed lines represent assay cut-off values determined by the mean plus three standard deviations of the negative control (BSA coupled beads). Statistics by standard two-way ANOVA. ****p-value < 0.0001.

Neutralizing antibodies of SARS-CoV-2 largely target the receptor binding domain present within the Spike S1 protein (24). Therefore, BALB/c mice were given two doses of commercially available Spike S1, 21 days apart (**Figure 1A**). To test whether adjuvants may alter immunological responses to the immunogen, mice were divided into four groups based on vaccine formulation. The mice receiving S1 protein and CoVaccine HT™ (S1+CoVac), Alhydrogel (S1+Alum), or PBS (S1+PBS) received SARS-CoV-2 Spike S1 mixed with either CoVaccine HT™ (“CoVac”), Alhydrogel (“Alum”), or PBS, respectively. One group received CoVaccine HT™ alone as an adjuvant control (**Figure 1A**).

### Adjuvants Alter Immunogenicity and Specificity to Immunization

Serum analysis revealed high reactivity of SARS-CoV-2 S1 specific IgG antibodies in S1+CoVac after a single dose while S1+Alum titres were near baseline (**Figure 1B**). Only one animal showed a detectable titre in the antigen alone group at this time point. Only in the group with CoVaccine HT™ a low level of cross reactivity was observed after the first dose to SARS-CoV S1. On day 35, S1+Alum and S1+PBS displayed significantly higher antibody responses compared to day 14 and variations among individual animals were reduced. S1+CoVac treated animals on day 35 consistently showed very high antibody responses in every animal. Similarly, cross-reactivity with SARS-CoV S1 was greatly increased for all groups on day 35 (**Figure 1B**). As expected, due to its much lower sequence homology, the SARS-CoV-2 S1 did not induce IgG responses to MERS-CoV S1.

In patients suffering from COVID-19, high RBD-specific IgG titres have been observed (25). However, higher titres of SARS-CoV-2 Spike-specific IgG are associated with patients that did not require intensive care unit treatment while lower titres are associated with increased disease severity (26). Therefore, the antibody response kinetics may be an important factor for a successful vaccine candidate. Time-course analysis of IgG responses reveal that adjuvanted S1 may be crucial for strong, early IgG responses with SARS-CoV-2 specificity while a second dose may decrease variability among individual animals and increase cross-reactivity (**Figure 1C**).

### CoVaccine HT™ Improves IgG Titres to SARS-CoV-2 and SARS-CoV S1 Proteins

**Figure 2:**
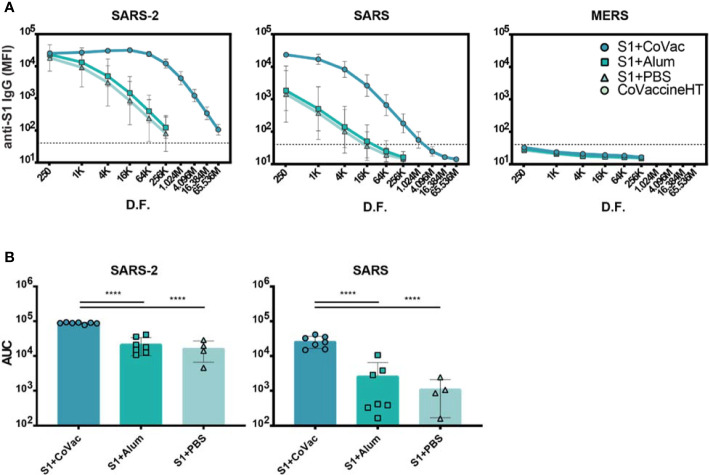
Serum IgG titres against Coronavirus S1 antigens. **(A)** Antigen reactivity in a four-fold dilution series of mouse sera. **(B)** Area under the curve (AUC) of data in **(A)**. Both graphs are in log scale with geometric mean and 95% CI. The dashed lines in **(A)** represent the cut-off value determined by the mean plus three standard deviations of the negative control (BSA coupled beads). K= x1000, M= x1,000,000, D.F.= dilution factor. Statistics by standard one-way ANOVA. ****p-value < 0.0001.

To further investigate the matured IgG responses, sera from day 35 were titrated in a four-fold dilution series starting at 1/250 and analysed by microsphere immunoassay (MIA). The S1+Alum and S1+PBS groups showed reactivity to SARS-CoV-2 S1 when diluted up to 1/256,000, indicating an abundance of antigen-specific IgG in the sera (**Figure 2A**). Titrating sera from S1+CoVac however, revealed saturating levels of IgG for five dilutions and detectable IgG levels were present down to a 1/65.5 million dilution. Antiserum to S1+CoVac also showed significantly greater cross reactivity to SARS-CoV S1 compared to the other groups. All groups remained negative for cross reactivity to MERS-CoV S1 (**Figure 2A**). These data suggest that immunization with SARS-CoV-2 S1 and CoVaccine HT™ elicits robust antigen-specific IgG response with the expected cross-reactivity profile to include SARS-CoV S1 (**Figure 2B**).

### Increased IgG Subclass Diversity and Enhanced Viral Neutralizing Antibody Titres With CoVaccine HT™

**Figure 3:**
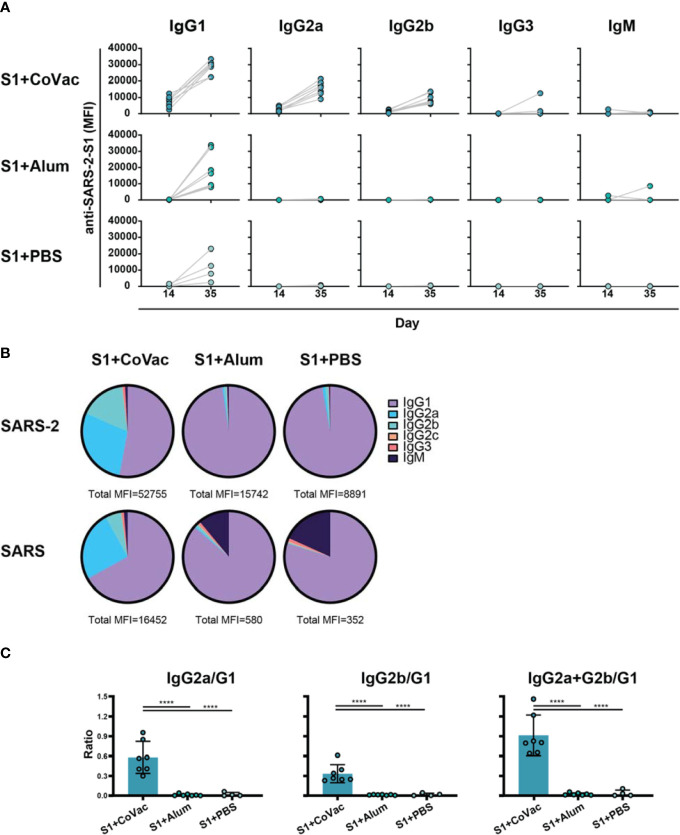
Adjuvant effects on immunoglobulin subclass diversity. **(A)** IgG subclasses reacting with SARS-2 S1 antigen between day 14 and day 35 plotted on a linear scale. **(B)** Relative abundance of Immunoglobulin isotypes and IgG subclasses reacting to SARS-2 and SARS antigens determined by subtracting the specified subclass cut-off values from the geometric mean of each group. The total median fluorescence intensity (MFI) from which the subclasses are a fraction of is listed below each pie-chart. **(C)** Ratios of subclasses. The normalized MFI values of each subclass per mouse were plotted as ratios using mean and SD. Statistics by standard one-way ANOVA. ****p-value < 0.0001.

Adjuvants serving as TLR4 agonists, such as postulated for CoVaccine HT™, elicit a primarily T_h_1 type response (27, 28). Meanwhile, Alhydrogel facilitates a mainly T_h_2 type response, possibly through NOD-like receptor signalling (29, 30). IgG subclass analysis can be used to determine if a T_h_1 or T_h_2 response may have been more prominent. Therefore, sera from each S1+adjuvant group were analysed for their subclass composition (**Figure 3**). Consistent with previous findings, the S1+CoVac group displayed a diverse immunoglobulin response composed of IgG1, IgG2a, and IgG2b subclasses all of which were further elevated after a second dose of vaccine. Low levels of IgG3 were also observed. Alternatively, the Alum and antigen alone groups primarily produced an IgG1 response with some detectable IgM in the Alum group, representing a classical T_h_2-biased humoral response. Heterogeneous subclass populations such as those observed in the S1+CoVac group are typically associated with T_h_1 responses while IgG1 is characteristic of a T_h_2 response. To further investigate the nature of these adjuvant effects, the subclass data were stratified to analyse ratios of T_h_1 vs T_h_2 subclasses (**Figure 3C**). This analysis clearly shows that of the three tested formulations, only S1+CoVac induced a relatively balanced humoral response. Furthermore, only the S1+CoVac formulation was able to induce detectable SARS-CoV-2 neutralizing antibody titres as demonstrated in a plaque reduction neutralization test using wildtype virus (**Table 1**). PRNT_90_ and PRNT_50_ titres for this formulation indicate potent neutralization (1:1620).

**Table 1:** SARS-CoV-2 neutralization titres.

Group ID	Titre (PRNT_90_)	Titre (PRNT_50_)
*S1+CoVac*	1620	1620
*S1+Alum*	<20	<20
*S1+PBS*	<20	<20
*CoVaccine HT*	<20	<20

### Adjuvant Effect on the SARS-CoV-2 S1-Specific IFN-γ Responses

**Figure 4:**
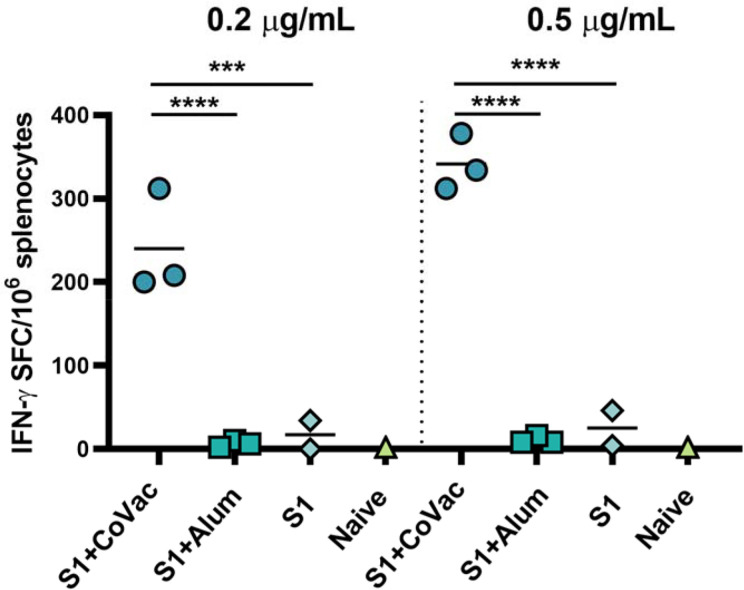
Detection of IFN-γ secreting cells from mice immunized with SARS-CoV-2. vaccines. The splenocytes were obtained from mice (2 to 3 per group) immunized with SARS-CoV-2 S1 protein, adjuvanted with CoVaccine HT™ or Alum, or S1 protein alone on day 28 (one-week after booster immunizations). Pooled splenocytes obtained from two naïve mice were used as controls. The cells were incubated for 40 h with PepTivator^®^ SARS-CoV-2 Prot_S1 peptide pools at 0.2 μg/ml or 0.5 μg/ml per peptide or medium. IFN-γ secreting cells were enumerated by FluoroSpot as detailed in the methods section. The results are expressed as the number of spot forming cells (SFC)/10^6^ splenocytes after subtraction of the number of spots formed by cells in medium only wells to correct for background activity. Significance of differences between groups was determined by one-way ANOVA followed by a “Tukey’s multiple comparison” ***p ≤ 0.001, ****p ≤ 0.0001.

We assessed the adjuvant effect of CoVaccine HT™ and Alum on the cellular immune responses directed against SARS-CoV-2 S1 using an IFN-γ FluoroSpot assay. Individual mouse spleens from each group harvested at day 7 post-second immunization were processed, and single cell suspensions stimulated with SARS-CoV-2 S1 peptides. The number of IFN-γ secreting cells from the mice given CoVaccine HT™ was significantly higher than those for mice given Alum or S1 antigen only at two different peptide concentrations (**Figure 4**). Splenocytes from unvaccinated (naïve) mice did not respond to S1 peptide stimulation with only 2 spot forming cells (SFCs)/10 (6) cells detected. The results suggest that CoVaccine HT™ is a superior adjuvant for induction of an antigen-specific Th1-focused cellular immune response, which is critical for SARS-CoV-2 vaccine development.

## Discussion

The COVID-19 pandemic has stimulated global efforts to rapidly develop vaccines against SARS-CoV-2. Many vaccine strategies are being explored, including inactivated virus, non-replicating viral vectors, recombinant protein, DNA, and RNA several of which have reached human clinical trials (6, 7). The number of clinically applied adjuvants are limited and include Alum and newer formulations such as MF59 and AS03, both O/W emulsions using squalene (31). The small number of adjuvants approved for clinical use has limited vaccine development in the past and impacts current clinical trials of SARS-CoV-2 vaccines. Many approaches use no adjuvant, Alum, MF59, or AS03, however, Novavax is testing the experimental adjuvant Matrix-M™ (32). While Alum is known to primarily enhance a T_h_2 response, Matrix-M™ and CoVaccine HT™ have both been shown to elicit a T_h_1 response with recombinant subunits. Due to the previously observed potential for enhanced immunopathology associated with primarily T_h_2-targeted anti-SARS-CoV vaccines (33, 34), the development of a COVID-19 vaccine may require testing of a multitude of adjuvants to elicit protective immune responses to SARS-CoV-2. The squalane-in-water based adjuvant, CoVaccine HT™, has previously been shown to induce potent virus neutralization antibody titres and protective efficacy in mice and non-human primates to several infectious agents, and has recently been licensed by Soligenix, Inc. for use in SARS-CoV-2 vaccine development (35–38).

In this study, we investigated the immunogenicity of recombinant SARS-CoV-2 Spike S1 alone or in combination with Alum or CoVaccine HT™ as potential adjuvants. Overall, we observed the most potent humoral and cellular immune responses, including neutralizing antibody responses in the CoVaccine HT™ study group. Day 14 titres (post-dose 1) indicate that this formulation may even be efficacious after administration of a single dose, however, this was not investigated in the current study. Neither antigen alone nor the combination with Alum was able to induce detectable neutralizing antibodies with the model antigen utilized in this study. This may be due to slower response kinetics caused by antigen presentation, subclass homogeneity, or T_h_2 restricted immune responses compared to administering S1 with CoVaccine HT™.

Immunogenicity of protein subunit vaccines is often inferior in generating robust immune responses compared to other platforms such as those based on live attenuated viruses. As seen here, the (monomeric) S1 domain alone is not adequate for generating a high titre immune response. The addition of CoVaccine HT™ improved antibody titres and response kinetics and proved to induce high titres of antibodies neutralizing wild-type SARS-CoV-2. It has been shown by others that SARS-CoV-2 S1 IgG titres correlate with viral neutralization in humans (39). Virus neutralizing responses in rabbits after two immunizations with 50 µg of SARS-CoV-2 S1 and Emulsigen adjuvant (O/W emulsion) were at 1:160 in the wild-type neutralization assay, compared to titres at 1:800 achieved when immunising with SARS-CoV-2 RBD (40). Similarly, emulsion-based adjuvants improved kinetics and antibody titres in guinea pigs compared to Alhydrogel or no adjuvant with HIV-1 gp140 immunization (41). This demonstrates the importance of achieving an antigen/adjuvant combination with desirable properties. In our study, post-dose 1 titres in the S1+CoVac group resemble post-dose 2 titres with Alum or no adjuvant and may suggest at least partial protection after a single dose. Generating potent immunity after a single dose is an attractive target for any SARS-CoV-2 vaccine in development and may improve the impact of a vaccine on the further course of the pandemic.

The high potency for SARS-CoV-2 S1 in the CoVaccine HT™ formulation may be attributable to the observed immunoglobulin subclass diversity. This indicates CoVaccine HT™ may efficiently induce class switching often considered to increase antibody affinity. Furthermore, a broad IgG subclass composition is key for inducing complement-mediated antibody effector functions as well as neutralization and opsonization, which are typically essential for mitigating viral infections. The ideal antibody population has yet to be elucidated for combating SARS-CoV-2. However, our murine serological data suggests kinetics and subclass diversity may be key to developing effective immune responses. Additionally, we have demonstrated that CoVaccine HT™ is not only a suitable adjuvant for vaccination but is preferable to Alhydrogel given the quality of the humoral response due to rapid onset, balance, overall magnitude of the response, as well as significantly greater cell-mediated immune responses.

Concerns have been raised regarding antibody dependent enhancement (ADE) with SARS-CoV-2 infection or immunization. This phenomenon occurs when non-neutralizing or poorly binding antibodies interact with Fc receptors on antigen presenting cells and facilitate infection. This interaction increases pro-inflammatory cytokine production which exacerbates immunopathology (42). ADE was previously observed with SARS-CoV infection caused by anti-Spike antibodies through the FcγR and FcγRII pathways (43, 44). In respiratory syncytial virus infections, a T_h_2 response alone can lead to aberrant immune responses associated with ADE caused by either a prior infection or immunization (45, 46). For these reasons, caution may be warranted when using Alum as an adjuvant for SARS-CoV-2, at least for the subunit protein used in this study. Additionally, our data suggest that antigen alone may also drive a predominantly T_h_2 type response with recombinant S1 antigen immunization in mice. In contrast, the subunit protein with CoVaccine HT™ produces neutralizing antibodies while boosting T_h_1 responses which may increase durability, vaccine safety, and efficacy.

Previous studies of SARS-CoV, the most closely related human betacoronavirus to SARS-CoV-2, have shown that recovered patients develop substantial T cell responses, which persist for up to 11 years (47, 48). In addition, animal studies indicated that T cell responses play a crucial role in protection against SARS-CoV infection (49–51), suggesting that it is most likely that T cell responses to SARS-CoV-2 are protective. Despite very limited understanding on the adaptive immune responses to SARS-CoV-2, it has been reported that virus-specific T cell responses are detected in 70% to 100% of COVID-19 convalescent patients and about 50% of the CD4+ T cell response is directed against S protein and correlates with the magnitude of anti-S antibody response (52). Furthermore, the majority of CD4^+^ T cells appears to be T_h_1 type with little or no T_h_2 cytokine secretion detected (52). This suggests that a SARS-CoV-2 vaccine candidate consisting of S protein can induce a robust CD4^+^ T cell response that recapitulates the elicited immune response during the natural infection. In this study, we showed that SARS-CoV-2 S1 adjuvanted with CoVaccine HT™ elicits a strong IFN-γ secreting cellular immune response upon peptide stimulation, indicating the induction of a T_h_1-targeted T cell response, which highlights the potential of this antigen/adjuvant combination to protect against SARS-CoV-2 infection. However, further in-depth analysis of cellular immune responses will be needed to characterize CD4^+^ and CD8^+^ T cell responses and their correlation with the antibody titres. Future work should also investigate mechanisms of action on a cellular level and potentially include a larger selection of O/W adjuvants to delineate differences of potential clinical relevance.

Altogether, CoVaccine HT™ is an effective adjuvant that in combination with a properly chosen recombinant subunit protein promotes rapid induction of balanced humoral and cellular immune responses and allows accelerated preclinical and clinical development of a SARS-CoV-2 vaccine to mitigate the ongoing COVID-19 pandemic.

## Data Availability Statement

The raw data supporting the conclusions of this article will be made available by the authors upon request, without undue reservation.

## Ethics Statement

The animal study was reviewed and approved by University of Hawaii Institutional Animal Care and Use Committee.

## Author Contributions

Authors BH and C-YL have equal contribution. BH, CW, TW, C-YL, ML, and AL: Conceived and designed the experiments. BH and TW: Immunizations and blood collection. BH and C-YL: Splenectomy. BH: Microsphere immunoassays and Flow cytometry studies. C-YL: Fluorospot assay. BIOQUAL: Virus neutralization assays. BH, CW, TW, ML, and AL: Manuscript writing and editing. All authors contributed to the article and approved the submitted version.

## Funding

We would like to acknowledge partial funding for these studies from R01AI132323 (National Institute of Allergy and Infectious Diseases), from P30GM114737 (Centers of Biomedical Research Excellence, National Institute of General Medical Sciences) and institutional funds. The funders played no role in the conceptualization, design, data collection, analysis, decision to publish, or preparation of the manuscript.

## Conflict of Interest

AL is named on a patent application covering a recombinant subunit vaccine for SARS-CoV-2. LP and HA-E were employed by BIOQUAL, Inc.

The remaining authors declare that the research was conducted in the absence of any commercial or financial relationships that could be construed as a potential conflict of interest.

## References

[1] WHO Coronavirus disease (COVID-19) - Weekly Epidemiological Update. Geneva: World Health Organization (2020). p. 155 Available from: https://www.who.int/docs/default-source/coronaviruse/situation-reports/20200928-weekly-epi-update.pdf?sfvrsn=9e354665_6 (September 29, 2020).

[2] Wang Y Wang Y Chen Y Qin Q Unique epidemiological and clinical features of the emerging 2019 novel coronavirus pneumonia (COVID-19) implicate special control measures. J Med Virol (2020) 92(6):568–76. 10.1002/jmv.25748 PMC7228347 32134116

[3] Hoffmann M Kleine-Weber H Schroeder S Kruger N Herrler T Erichsen S SARS-CoV-2 Cell Entry Depends on ACE2 and TMPRSS2 and Is Blocked by a Clinically Proven Protease Inhibitor. Cell (2020) 181(2):271–80 e8. 10.1016/j.cell.2020.02.052 PMC7102627 32142651

[4] Tai W He L Zhang X Pu J Voronin D Jiang S Characterization of the receptor-binding domain (RBD) of 2019 novel coronavirus: implication for development of RBD protein as a viral attachment inhibitor and vaccine. Cell Mol Immunol (2020) 17(6):613–20. 10.1038/s41423-020-0400-4 PMC7091888 32203189

[5] Bisht H Roberts A Vogel L Subbarao K Moss B Neutralizing antibody and protective immunity to SARS coronavirus infection of mice induced by a soluble recombinant polypeptide containing an N-terminal segment of the spike glycoprotein. Virology (2005) 334(2):160–5. 10.1016/j.virol.2005.01.042 PMC7111832 15780866

[6] Thanh Le T Andreadakis Z Kumar A Gomez Roman R Tollefsen S Saville M The COVID-19 vaccine development landscape. Nat Rev Drug Discov (2020) 19(5):305–6. 10.1038/d41573-020-00073-5 32273591

[7] Milken-Institute COVID-19 Treatment and Vaccine Tracker. Santa Monica: Milken Institute (2020). Available at: https://covid-19tracker.milkeninstitute.org/#vaccines_intro (September 29, 2020).

[8] Stevens NE Fraser CK Alsharifi M Brown MP Diener KR Hayball JD An empirical approach towards the efficient and optimal production of influenza-neutralizing ovine polyclonal antibodies demonstrates that the novel adjuvant CoVaccine HT is functionally superior to Freund’s adjuvant. PLoS One (2013) 8(7):e68895. 10.1371/journal.pone.0068895 23894371 PMC3720891

[9] Blom AG Hilgers LA Sucrose fatty acid sulphate esters as novel vaccine adjuvants: effect of the chemical composition. Vaccine (2004) 23(6):743–54. 10.1016/j.vaccine.2004.07.021 15542198

[10] Harris JR Soliakov A Lewis RJ Depoix F Watkinson A Lakey JH Alhydrogel(R) adjuvant, ultrasonic dispersion and protein binding: a TEM and analytical study. Micron (2012) 43(2–3):192–200. 10.1016/j.micron.2011.07.012 21831642

[11] Sun H Pollock KG Brewer JM Analysis of the role of vaccine adjuvants in modulating dendritic cell activation and antigen presentation *in vitro* . Vaccine (2003) 21(9–10):849–55. 10.1016/s0264-410x(02)00531-5 12547593

[12] Snell LM Osokine I Yamada DH De la Fuente JR Elsaesser HJ Brooks DJ Overcoming CD4 Th1 Cell Fate Restrictions to Sustain Antiviral CD8 T Cells and Control Persistent Virus Infection. Cell Rep (2016) 16(12):3286–96. 10.1016/j.celrep.2016.08.065 PMC5669380 27653690

[13] To A Medina LO Mfuh KO Lieberman MM Wong TAS Namekar K Recombinant Zika Virus Subunits Are Immunogenic and Efficacious in Mice. mSphere (2018) 3e00576-17. 10.1128/mSphere.00576-17 PMC5760751 29359186

[14] Orr MT Khandhar AP Seydoux E Liang H Gage E Mikasa T Reprogramming the adjuvant properties of aluminum oxyhydroxide with nanoparticle technology. NPJ Vaccines (2019) 4:1. 10.1038/s41541-018-0094-0 30622742 PMC6318334

[15] Hilgers LAT Platenburg PL II Luitjens A Groenveld B Dazelle T Ferrari-Laloux M A novel non-mineral oil-based adjuvant. I. Efficacy of a synthetic sulfolipopolysaccharide in a squalane-in-water emulsion in laboratory animals. Vaccine (1994) 12(7):653–60. 10.1016/0264-410x(94)90272-0 8085385

[16] Hilgers LA Blom AG Sucrose fatty acid sulphate esters as novel vaccine adjuvant. Vaccine (2006) 24 Suppl 2:S2–81-82. 10.1016/j.vaccine.2005.01.133 16823938

[17] Shah RR Taccone M Monaci E Brito LA Bonci A O’Hagan DT The droplet size of emulsion adjuvants has significant impact on their potency, due to differences in immune cell-recruitment and -activation. Sci Rep (2019) 9(1):11520. 10.1038/s41598-019-47885-z 31395915 PMC6687744

[18] Allison AC Squalene and squalane emulsions as adjuvants. Methods (1999) 19(1):87–93. 10.1006/meth.1999.0832 10525443

[19] Garcon N Vaughn DW Didierlaurent AM Development and evaluation of AS03, an Adjuvant System containing alpha-tocopherol and squalene in an oil-in-water emulsion. Expert Rev Vaccines (2012) 11(3):349–66. 10.1586/erv.11.192 22380826

[20] Namekar M Kumar M O’Connell M Nerurkar VR Effect of serum heat-inactivation and dilution on detection of anti-WNV antibodies in mice by West Nile virus E-protein microsphere immunoassay. PLoS One (2012) 7(9):e45851. 10.1371/journal.pone.0045851 23049879 PMC3457982

[21] Wong SJ Demarest VL Boyle RH Wang T Ledizet M Kar K Detection of human anti-flavivirus antibodies with a west nile virus recombinant antigen microsphere immunoassay. J Clin Microbiol (2004) 42(1):65–72. 10.1128/JCM.42.1.65-72.2004 14715733 PMC321652

[22] Haun BK Kamara V Dweh AS Garalde-Machida K Forkay SSE Takaaze M Serological evidence of Ebola virus exposure in dogs from affected communities in Liberia: A preliminary report. PLoS Neglected Trop Dis (2019) 13(7):e0007614. 10.1371/journal.pntd.0007614 PMC6684096 31329600

[23] Kumar M O’Connell M Namekar M Nerurkar VR Infection with non-lethal West Nile virus Eg101 strain induces immunity that protects mice against the lethal West Nile virus NY99 strain. Viruses (2014) 6(6):2328–39. 10.3390/v6062328 PMC4074930 24915459

[24] Jiang S Hillyer C Du L Neutralizing Antibodies against SARS-CoV-2 and Other Human Coronaviruses: (Trends in Immunology 41, 355-359; 2020). Trends Immunol (2020) 41(6):545. 10.1016/j.it.2020.04.008 32249063 PMC7129017

[25] Ma H Zeng W He H Zhao D Jiang D Zhou P Serum IgA, IgM, and IgG responses in COVID-19. Cell Mol Immunol (2020) 17(7):773–5. 10.1038/s41423-020-0474-z PMC7331804 32467617

[26] Sun B Feng Y Mo X Zheng P Wang Q Li P Kinetics of SARS-CoV-2 specific IgM and IgG responses in COVID-19 patients. Emerg Microbes Infect (2020) 9(1):940–8. 10.1080/22221751.2020.1762515 PMC7273175 32357808

[27] Matsuoka Y Takagi H Yamatani M Kuroda Y Sato K Kojima N Requirement of TLR4 signaling for the induction of a Th1 immune response elicited by oligomannose-coated liposomes. Immunol Lett (2016) 178:61–7. 10.1016/j.imlet.2016.07.016 27485186

[28] Perrin-Cocon L Agaugue S Diaz O Vanbervliet B Dollet S Guironnet-Paquet A Th1 disabled function in response to TLR4 stimulation of monocyte-derived DC from patients chronically-infected by hepatitis C virus. PLoS One (2008) 3(5):e2260. 10.1371/journal.pone.0002260 18509450 PMC2377338

[29] Li H Willingham SB Ting JP Re F Cutting edge: inflammasome activation by alum and alum’s adjuvant effect are mediated by NLRP3. J Immunol (2008) 181(1):17–21. 10.4049/jimmunol.181.1.17 18566365 PMC2587213

[30] Morefield GL Sokolovska A Jiang D HogenEsch H Robinson JP Hem SL Role of aluminum-containing adjuvants in antigen internalization by dendritic cells *in vitro* . Vaccine (2005) 23(13):1588–95. 10.1016/j.vaccine.2004.07.050 15694511

[31] CDC What is an adjuvant and why is it added to a vaccine? Atlanta: Centers for Disease Control and Prevention (2020). Available at: https://www.cdc.gov/vaccinesafety/concerns/adjuvants.html (October 24, 2018).

[32] Gupta T Gupta SK Potential adjuvants for the development of a SARS-CoV-2 vaccine based on experimental results from similar coronaviruses. Int Immunopharmacol (2020) 86:106717. 10.1016/j.intimp.2020.106717 32585611 PMC7301105

[33] Luo F Liao FL Wang H Tang HB Yang ZQ Hou W Evaluation of Antibody-Dependent Enhancement of SARS-CoV Infection in Rhesus Macaques Immunized with an Inactivated SARS-CoV Vaccine. Virol Sin (2018) 33(2):201–4. 10.1007/s12250-018-0009-2 PMC6178114 29541941

[34] Tseng CT Sbrana E Iwata-Yoshikawa N Newman PC Garron T Atmar RL Immunization with SARS coronavirus vaccines leads to pulmonary immunopathology on challenge with the SARS virus. PLoS One (2012) 7(4):e35421. 10.1371/journal.pone.0035421 22536382 PMC3335060

[35] Medina LO To A Lieberman MM Wong TAS Namekar M Nakano E A Recombinant Subunit Based Zika Virus Vaccine Is Efficacious in Non-human Primates. Front Immunol (2018) 9:2464. 10.3389/fimmu.2018.02464 30467501 PMC6236113

[36] Lehrer AT Wong TS Lieberman MM Humphreys T Clements DE Bakken RR Recombinant proteins of Zaire ebolavirus induce potent humoral and cellular immune responses and protect against live virus infection in mice. Vaccine (2018) 36(22):3090–100. 10.1016/j.vaccine.2017.01.068 PMC8426131 28216187

[37] Mahdi Abdel Hamid M Remarque EJ van Duivenvoorde LM van der Werff N Walraven V Faber BW Vaccination with Plasmodium knowlesi AMA1 formulated in the novel adjuvant co-vaccine HT protects against blood-stage challenge in rhesus macaques. PLoS One (2011) 6(5):e20547. 10.1371/journal.pone.0020547 21655233 PMC3105089

[38] Kusi KA Remarque EJ Riasat V Walraven V Thomas AW Faber BW Safety and immunogenicity of multi-antigen AMA1-based vaccines formulated with CoVaccine HT and Montanide ISA 51 in rhesus macaques. Malar J (2011) 10:182. 10.1186/1475-2875-10-182 21726452 PMC3142537

[39] Robbiani DF Gaebler C Muecksch F Lorenzi JCC Wang Z Cho A Convergent antibody responses to SARS-CoV-2 in convalescent individuals. Nature (2020) 584(7821)437–42. 10.1038/s41586-020-2456-9 PMC7442695 32555388

[40] Ravichandran S Coyle EM Klenow L Tang J Grubbs G Liu S Antibody signature induced by SARS-CoV-2 spike protein immunogens in rabbits. Sci Transl Med (2020) 12(550)eabc3539. 10.1126/scitranslmed.abc3539 PMC7286538 32513867

[41] Nkolola JP Cheung A Perry JR Carter D Reed S Schuitemaker H Comparison of multiple adjuvants on the stability and immunogenicity of a clade C HIV-1 gp140 trimer. Vaccine (2014) 32(18):2109–16. 10.1016/j.vaccine.2014.02.001 PMC3980048 24556505

[42] Iwasaki A Yang Y The potential danger of suboptimal antibody responses in COVID-19. Nat Rev Immunol (2020) 20(6):339–41. 10.1038/s41577-020-0321-6 PMC7187142 32317716

[43] Jaume M Yip MS Cheung CY Leung HL Li PH Kien F Anti-severe acute respiratory syndrome coronavirus spike antibodies trigger infection of human immune cells *via a* pH- and cysteine protease-independent FcgammaR pathway. J Virol (2011) 85(20):10582–97. 10.1128/JVI.00671-11 PMC3187504 21775467

[44] Yip MS Leung HL Li PH Cheung CY Dutry I Li D Antibody-dependent enhancement of SARS coronavirus infection and its role in the pathogenesis of SARS. Hong Kong Med J (2016) 22(3 Suppl 4):25–31. 27390007

[45] Graham BS Henderson GS Tang YW Lu X Neuzil KM Colley DG Priming immunization determines T helper cytokine mRNA expression patterns in lungs of mice challenged with respiratory syncytial virus. J Immunol (1993) 151(4):2032–40. 8345194

[46] Kim HW Canchola JG Brandt CD Pyles G Chanock RM Jensen K Respiratory syncytial virus disease in infants despite prior administration of antigenic inactivated vaccine. Am J Epidemiol (1969) 89(4):422–34. 10.1093/oxfordjournals.aje.a120955 4305198

[47] Li CK Wu H Yan H Ma S Wang L Zhang M T cell responses to whole SARS coronavirus in humans. J Immunol (Baltimore Md: 1950) (2008) 181(8):5490–500. 10.4049/jimmunol.181.8.5490 PMC2683413 18832706

[48] Ng OW Chia A Tan AT Jadi RS Leong HN Bertoletti A Memory T cell responses targeting the SARS coronavirus persist up to 11 years post-infection. Vaccine (2016) 34(17):2008–14. 10.1016/j.vaccine.2016.02.063 PMC7115611 26954467

[49] Chen J Lau YF Lamirande EW Paddock CD Bartlett JH Zaki SR Cellular immune responses to severe acute respiratory syndrome coronavirus (SARS-CoV) infection in senescent BALB/c mice: CD4+ T cells are important in control of SARS-CoV infection. J Virol (2010) 84(3):1289–301. 10.1128/jvi.01281-09 PMC2812346 19906920

[50] Zhao J Zhao J Perlman S T cell responses are required for protection from clinical disease and for virus clearance in severe acute respiratory syndrome coronavirus-infected mice. J Virol (2010) 84(18):9318–25. 10.1128/jvi.01049-10 PMC2937604 20610717

[51] Channappanavar R Fett C Zhao J Meyerholz DK Perlman S Virus-specific memory CD8 T cells provide substantial protection from lethal severe acute respiratory syndrome coronavirus infection. J Virol (2014) 88(19):11034–44. 10.1128/jvi.01505-14 PMC4178831 25056892

[52] Grifoni A Weiskopf D Ramirez SI Mateus J Dan JM Moderbacher CR Targets of T Cell Responses to SARS-CoV-2 Coronavirus in Humans with COVID-19 Disease and Unexposed Individuals. Cell (2020) 181(7):1489–1501.e1415. 10.1016/j.cell.2020.05.015 32473127 PMC7237901

[53] Haun BK Lai CY Williams CA Wong TA Lieberman MM Pessaint L CoVaccine HT adjuvant potentiates robust immune responses to recombinant SARS-CoV-2 Spike S1 immunisation. bioRxiv (2020). 10.1101/2020.07.24.220715 PMC7661386 33193454

